# Callous-unemotional traits moderate executive function in children with ASD and ADHD: A pilot event-related potential study

**DOI:** 10.1016/j.dcn.2017.06.002

**Published:** 2017-06-13

**Authors:** C. Tye, R. Bedford, P. Asherson, K.L. Ashwood, B. Azadi, P. Bolton, G. McLoughlin

**Affiliations:** a King’s College London, MRC SGDP Centre, Institute of Psychiatry, Psychology & Neuroscience, United Kingdom; b King’s College London, Child & Adolescent Psychiatry, Institute of Psychiatry, Psychology & Neuroscience, United Kingdom; c King’s College London, Department of Biostatistics, Institute of Psychiatry, Psychology & Neuroscience, United Kingdom; d King’s College London, Department of Psychology, Institute of Psychiatry, Psychology & Neuroscience, United Kingdom

**Keywords:** Autism, Attention deficit hyperactivity disorder, Callous unemotional traits, Executive function, Event-related potentials (ERPs)

## Abstract

Attention deficit hyperactivity disorder (ADHD) and autism spectrum disorder (ASD) are associated with varied executive function (EF) difficulties. Callous-unemotional (CU) traits, a proposed antecedent of adult psychopathy, are often associated with intact or enhanced EF. Here we test whether CU traits may therefore modulate EF in ASD and ADHD, in which EF is typically impaired. We collected CU traits and measured event-related potentials (ERPs) that index EF during a cued-continuous performance test (CPT-OX) in boys with ASD, ADHD, comorbid ASD + ADHD and typical controls. We examined attentional orienting at cues (Cue-P3), inhibitory processing at non-targets (NoGo-P3) and conflict monitoring between target and non-target trials (Go-N2 vs. NoGo-N2). In children with ASD, higher CU traits were associated with an enhanced increase in N2 amplitude in NoGo trials compared to Go trials, which suggests relatively superior conflict monitoring and a potential cognitive strength associated with CU traits. The results emphasise the importance of considering the effects of co-occurring traits in the assessment of heterogeneity of EF profiles in neurodevelopmental disorders.

## Highlights

• Children with ASD and ADHD show varied and heterogeneous executive function (EF) profiles.
• Typical or enhanced EF has been demonstrated in individuals with callous-unemotional (CU) traits.
• We investigated the effect of CU traits on event-related potential (ERP) responses during a cued continuous performance test (CPT-OX) in children with ASD, ADHD and co-occurring ASD + ADHD.
• Children with ASD and high CU traits showed better conflict monitoring compared to children with ASD and low CU traits.
• Increased CU traits may be associated with cognitive strengths in children with ASD.

## 1 Introduction

Autism spectrum disorder (ASD) and attention deficit hyperactivity disorder (ADHD) are two common childhood-onset disorders that show substantial behavioural and genetic overlap (Ronald et al., 2008). Impairments in executive function (EF), behaviours such as planning, online monitoring and working memory, characterise both children with ASD and ADHD, and may underlie some of the behavioural features of the disorders Happé et al., 2006, Rommelse et al., 2011). Children with ASD often perform poorly on tasks requiring planning and mental flexibility, while children with ADHD consistently demonstrate difficulties inhibiting responses (Geurts et al., 2004, Happé et al., 2006). Event-related potentials (ERPs) which capture distinct underlying neural processes related to these functions, have demonstrated that ASD and ADHD can be dissociated on the basis of their neurophysiological responses during attentional (Tye et al., 2014a) and social cognitive tasks (Tye et al., 2013, Tye et al., 2014b). Specifically, children with ADHD symptoms (both ADHD and comorbid ASD *and* ADHD; ASD + ADHD) demonstrate impairment in response inhibition (reduced NoGo-P3 to non-targets) and attentional orienting (reduced Cue-P3 to cue/warning stimuli), while children with ASD (ASD and ASD + ADHD) show reduced conflict monitoring (reduced N2 enhancement from Go (target) to NoGo (non-target) trials; Tye et al., 2014a), on a cued Continuous Performance Task (CPT-OX). These findings indicate that impaired EF processes are distinct in ASD and ADHD, whereas children with co-occurring ASD + ADHD present as an additive co-occurrence with the unique deficits of both disorders. Still, little is known about the role of other co-occurring traits in moderating EF in ASD and ADHD and their overlap, particularly those that are associated with typical EF. The recent shift toward dimensional over categorical approaches in psychopathology (Cuthbert and Insel, 2013) emphasises the importance of a transdiagnostic approach, assessing traits rather than categorical disorders. Linking neurocognitive markers to dimensions will likely be more informative in terms of understanding the underlying mechanisms.

There has been growing interest in the comorbidity demonstrated between psychopathic tendencies, anti-social behaviour and both ASD and ADHD (Colledge and Blair, 2001; Kadesjö and Gillberg, 2001; Simonoff et al., 2008; Leno et al., 2015). Children with ASD display increased antisocial and aggressive behaviour (Bauminger et al., 2010) and a quarter to a third of individuals have a co-occurring diagnosis of oppositional defiant disorder (ODD) and/or conduct disorder (CD; Simonoff et al., 2008, Kaat et al., 2013). These disruptive behaviours tend to have a highly stable and persistent course when left untreated and are associated with a higher rate of dysfunctional outcomes. Follow-up studies of children with ADHD indicate 21% meet criteria for antisocial personality disorder (ASPD) in young adulthood, with the severity of childhood conduct problems as a contributory factor (Fischer et al., 2002). Psychiatric comorbidity in ASD is a major factor contributing to violent offending (Woodbury-Smith et al., 2005, Newman and Ghaziuddin, 2008) and there are consistent associations between ASD traits and psychopathic traits (Soderstrom et al., 2005). A longitudinal study, however, suggested no risk for ASPD in adult patients with a childhood diagnosis of ASD (0%), but an increased risk for those with childhood-onset ADHD (30.9%) and ASD + ADHD (18.5%; Anckarsäter et al., 2006), although ASPD may be more common in pervasive developmental disorder-not otherwise specified (Hofvander et al., 2009). The pathophysiological mechanisms underlying the developmental trajectories to antisocial behaviour and psychopathic traits may be separable in ASD and ADHD.

Recent research has highlighted the role of callous-unemotional (CU) traits, characterised by a lack of guilt, remorse and empathy, in increased risk for persistent antisocial behaviour and adult psychopathy (Barry et al., 2000, Frick et al., 2003, Viding, 2004, Frick and White, 2008, Frick et al., 2014). Individuals with CU traits represent a putative subgroup of antisocial behaviour that show several distinct cognitive and emotional characteristics (Frick et al., 2008). At the behavioural and neural level, CU traits are associated with a selective impairment in affective processing, which can be differentiated from the social cognitive deficits observed in ASD (Jones et al., 2010, Schwenck et al., 2012, Wallace et al., 2012, Lockwood et al., 2013, O'Nions et al., 2014). Accordingly, a “double hit” hypothesis has been proposed whereby individuals with ASD and elevated CU traits exhibit the unique profiles that are independently associated with each disorder rather than being inherently related to the core symptom of ASD (Rogers et al., 2006). In support of distinct causal factors associated with ASD and CU traits, largely independent genetic and environmental influences have been reported for ASD and psychopathic and CU traits (Jones et al., 2009).

While limited research has been conducted specifically investigating EF in individuals with high CU traits, attentional processes and EF have been studied with relation to antisocial behaviour and psychopathic traits (Morgan and Lilienfeld, 2000, Blair, 2005, Blair and Mitchell, 2009). Here we focus on the EF domains indexed in the cued CPT-OX (Tye et al., 2014a). Studies that have assessed response inhibition directly have shown that adults with psychopathy or elevated psychopathic traits tend to make more commission errors than typical adults during Go/No-Go tasks (Lapierre et al., 1995, Munro et al., 2007, Sellbom and Verona, 2007) and show reduced amplitude of the N275 ERP to NoGo stimuli (Kiehl et al., 2000), which suggests an inhibitory deficit. In contrast, another study showed that psychopathic offenders demonstrate the typical increase in the frontal N2 from Go to NoGo trials (Munro et al., 2007), which suggests intact conflict monitoring. This is indirectly supported by typical or better performance by individuals with high psychopathic traits compared to typical controls on attentional set-shifting tasks, such as the Wisconsin Card Sorting Task (Lapierre et al., 1995, Ishikawa et al., 2001, Mitchell et al., 2002) and executive attention tasks that involve conflict or error monitoring (Lapierre et al., 1995, Mitchell et al., 2002, Blair et al., 2006, Bresin et al., 2014). There is no work directly investigating the association between psychopathy and attentional orienting, as measured in Tye et al. (2014a), but there is some evidence for weaker alerting during the Attentional Network Task, indexing preparedness to respond, demonstrated by reduced P1 amplitude (Racer et al., 2011).

There is evidence, therefore, for typical or even superior EF in individuals with high psychopathic traits (Moffitt, 2003, Blair, 2005), which suggests that in some instances CU traits in childhood may confer a advantage through relative cognitive strengths. The potential buffering effect of CU traits has been shown previously, whereby higher verbal intelligence (Loney et al., 1998) and better social problem-solving abilities (Waschbusch et al., 2007) are demonstrated in children with conduct problems and CU traits compared to children with conduct problems alone. In the current study, we examine the moderating effect of CU traits within an ASD/ADHD population that has impaired EF. Previous findings have suggested that individuals with ASD and CU traits or delinquency show impairments in emotion recognition, yet EF is unaffected (Woodbury-Smith et al., 2005, Rogers et al., 2006, Leno et al., 2015). High CU traits in ASD (and ADHD) may therefore offer particular cognitive strengths that are associated with distinct neurophysiological profiles.

The aim of this preliminary study is to provide proof-of-concept that abnormal EF in children with ASD and ADHD is conditional upon the level of CU traits, using the same sample and analyses described in Tye et al. (2014a). Specifically, we investigate whether EF is a relative cognitive strength in individuals with CU traits and ASD/ADHD, and whether this effect differs between ASD and ADHD. We focus here on sensitive ERP markers of EF to enable investigation of covert and distinct information processing stages, selected on the basis of previous findings indicating specificity to ASD or ADHD (Tye et al., 2014a). We investigated (1) associations between CU traits and attentional orienting, inhibitory control and conflict monitoring; and (2) the moderating role of CU traits on the association between ASD, ADHD and ERP-indexed EF.

## 2 Material and methods

### 2.1 Sample

The sample from Tye et al. (2014a) was used for these analyses. Nineteen male participants with ASD, 18 with ADHD, 29 with ASD and ADHD, and 26 typically developing controls (TDC) took part in the study. The age range was 8–13 years; there was no significant difference in age across groups (Table 1). All participants were required to have an IQ > 70, normal or corrected-to-normal vision, and not to be taking any medication except for stimulants (6 participants with ADHD, 6 participants with ASD + ADHD), which had to be interrupted 48 h prior to testing sessions. Exclusion criteria included non-fluent English, specific medical disorders, other comorbid psychiatric disorder including conduct disorder (not including ODD), history of traumatic brain injury and a diagnosis of epilepsy.

**Table 1:** Clinical and demographic characteristics.

	Diagnosis										
	TDC (*n* = 26)		ASD (*n* = 19)		ADHD (*n* = 18)		ASD + ADHD (*n* = 29)				
	Mean	SD	Mean	SD	Mean	SD	Mean	SD	F	p	Post-hoc
Age	10.56	1.79	11.69	1.70	10.48	1.91	10.53	1.69	2.20	0.093	n.s.d.
Verbal IQ	120.00	14.40	113.79	23.87	105.94	18.47	110.41	15.67	2.48	0.066	n.s.d.
Performance IQ	115.73	13.89	111.05	13.31	101.67	11.60	106.72	11.97	4.86	0.004	TD > ADHD
Full-scale IQ	120.04	13.42	115.68	15.73	104.11	14.23	109.72	13.41	5.31	0.002	TD > ASD + ADHD, ADHD
SCQ	3.88	3.54	20.11	6.42	10.89	5.36	24.79	5.71	81.12	<0.001	TD < ADHD < ASD < ASD + ADHD
Conners DSM-Inattentive	56.08	11.05	67.11	14.13	83.94	7.41	80.21	11.59	29.85	<0.001	TD < ASD < ASD + ADHD, ADHD
Conners DSM-Hyperactive	58.88	17.02	66.11	12.99	87.89	3.25	84.00	7.63	32.76	<0.001	TD, ASD < ASD + ADHD, ADHD
CU traits (ICU total score)	17.46	7.82	28.33	9.80	33.11	10.05	39.79	7.53	31.93	<0.001	TD < ASD, ADHD < ASD + ADHD
Conduct problems (SDQ)	1.16	1.46	1.82	1.70	4.72	1.64	4.10	2.34	16.48	<0.001	TD, ASD < ADHD, ASD + ADHD

n.s.d = non-significant difference.

The participants were recruited from out-patient neurodevelopmental clinics and local parent support groups in southeast London. All participants had a clinical diagnosis made according to ICD-10 criteria (autism, Aspergers syndrome, ADHD combined type) and then underwent systematic and rigorous clinical assessment to confirm pure or comorbid research diagnosis (see Tye et al., 2014a). All cases were initially evaluated with Conners’ 3rd Edition Parent Rating Scale short form (Conners, 2008) and Social Communication Questionnaire (SCQ; Rutter et al., 2003). Cases of ASD were diagnosed using the Autism Diagnostic Interview–Revised (ADI-R; modified criteria IMGSAC, 1998) and the Autism Diagnostic Observation Schedule (ADOS-G; Gotham et al., 2007). Cases of ADHD were diagnosed using Parent Account of Childhood Symptoms (PACS; Taylor et al., 1986), which has been extensively used by the IMAGE consortium (Chen et al., 2008). Co-morbid ASD + ADHD cases met full diagnostic criteria for ASD using the ADI-R/ADOS and full diagnostic criteria for ADHD using the PACS. Trained and research-reliable postgraduate researchers carried out the ASD and ADHD research diagnostic assessments. Two additional measures were administered to aid group classification and in-depth assessment where diagnostic classification was unclear: the Strengths and Difficulties Questionnaire (SDQ; Goodman, 1997) and Development and Wellbeing Assessment (DAWBA; Goodman et al., 2000). An experienced clinical academic (PB) reviewed the available data and decided on the ‘best estimate’ diagnosis using this multi-measure multi-informant approach, with greater weight given to clinical diagnosis, followed by ADI-R, PACS and DAWBA.

The TD group consisted of children recruited through local schools and forums. TD children were assessed with the SDQ, SCQ and Conners’ questionnaires and were not included if they had any psychiatric diagnosis (see Tye et al., 2014a).

Ethical considerations: A medical ethics committee approved the study protocol (REC Ref: 08/H0803/161). Written parental consent was given before the experiment began.

### 2.2 Task and stimuli

The cued-CPT, flanker version (Banaschewski et al., 2003, Doehnert et al., 2010, McLoughlin et al., 2010, Tye et al., 2014a), consists of a black letter array formed of a centre letter flanked on each side by distractor letters, presented in four identical blocks of 100 letter arrays each. Participants were instructed to ignore the distractor letters and attend only to the centre letter. There were 11 different centre letters (O, X, H, B, C, D, E, F, G, J and L) subtending approximately 0.5. Target centre letters ‘X’ and ‘O’ were flanked by the incompatible letter ‘O’ or ‘X’ and distractor letters were flanked by either ‘X’ or ‘O’. The letter arrays were presented briefly (150 ms) every 1.65 s in a pseudo-random sequence at the centre of a computer monitor at a viewing distance of 120 cm. The 80 cues (XOX) initiated 40 cue-target sequences (XOX-OXO) and 40 cue-nontarget sequences (e.g. XOX-XDX). In 40 trials, a distractor-X letter array (OXO) was not preceded by the cue and had to be ignored, along with any other irrelevant letters. Participants were instructed to respond only to cue-target sequences (XOX-OXO) by pressing a button as quickly as possible with the index finger of their preferred hand. The task was practised (24 trials including three cue-target and two cue-nontarget sequences), and comprehension ascertained based on correct performance prior to task onset. The duration of the task was 11 min. The flankered CPT-OX was administered after 6 min of resting EEG data recording as part of a larger test battery (not presented here) with a total duration of 70 min. Presentation of the tasks was ordered in the same way for each group to control for effects of practice and fatigue. Participants were seated on a height-adjustable chair in a video-monitored testing cubicle.

### 2.3 Other measures

Callous-unemotional traits were measured (n = 90) using the Inventory of Callous-Unemotional Traits (ICU; Frick, 2004), designed to provide a reliable, resourceful and valid evaluation of CU traits in youths. It consists of 24 items assessing uncaring, callous and unemotional behaviour, rated on a four-point Likert scale from: 0 (Not at all true) to 3 (Definitely true). The severity of conduct problems as a covariate was taken from the Conduct subscale of the Strengths and Difficulties Questionnaire (n = 74, SDQ; Goodman, 1997). IQ was assessed using four subtests (Block Design, Vocabulary, Matrix Reasoning and Similarities) of the Wechsler Abbreviated Scale of Intelligence (Wechsler, 1999).

### 2.4 Electrophysiological recording and analysis

EEG was recorded using a 62 active electrode recording system (ActiCap, Brain Products, Munich, Germany; extended 10–20 montage). The recording reference electrode was positioned at FCz. Vertical and horizontal electrooculograms (EOGs) were simultaneously recorded from electrodes above and below the left eye and at the outer canthi. The signal was digitized at 500 Hz sampling rate, stored and analysed offline.

Data were analysed in Brain Vision Analyzer (2.0; Brain Products, Munich, Germany). The signal was re-referenced offline to the average reference and downsampled to 256 Hz. We applied 0.1–30 Hz (24 dB/Oct) Butterworth filters. Ocular artifacts were removed from the data using biased infomax independent component analysis (ICA). The extracted independent components were manually inspected and ocular artifacts were removed by back-projection of all but those components. Remaining artifacts exceeding 200 μV peak-to-peak in any channel were rejected from the data. Baseline correction was performed using a 200 ms prestimulus reference period. Stimulus-locked epochs (peristimulus window from −200 to 1650 ms) were averaged for the following trial types: cue (trials to letter XOX); go (trials to OXOs preceded by XOX); no-go (trials to random target letters e.g., ODO following XOX). Averages contained at least 19 segments, only included trials with correct responses (Go) or correctly rejected trials (NoGo, Cue), and were free from residual artifacts.

ERP amplitudes were restricted to leads for which effects were expected to be largest, based on previous studies (Banaschewski et al., 2003, Banaschewski et al., 2004, Jonkman, 2006, Valko et al., 2009). The P3 was calculated as the mean amplitude in a 400–700 ms latency window, because the activity within this time window occurred over a long period making it difficult to identify one peak, as has been done in previous similar studies (Groom et al., 2010). The Cue-P3 and Go-P3 were measured at Pz, and the NoGo-P3 was measured at Cz, Cpz and Pz due to increased anteriorisation with increasing age (Jonkman, 2006, Valko et al., 2009). The N2 was scored as the maximal negative peak at Fz between 170–400 ms. Grand average ERPs and topographical maps are reported in Tye et al. (2014a) and descriptive statistics and figures are presented in the Supplementary Material (Table S1, Figs. S1 & S2).

### 2.5 Statistical analysis

Six children were excluded from analyses on the basis of extreme omission errors ( > 70%) indicating a lack of attention to task and/or poor understanding of task instructions that limited the number of segments for reliable ERP analysis (ADHD *n* = 2; ASD + ADHD *n* = 4). One TD participant was removed from analysis because of technical difficulties during recording and two additional ASD + ADHD participants were removed from the Go condition because of insufficient segments. Correlations between IQ and age and each of the ERP parameters were calculated across the whole sample, which indicated no significant associations (all p > 0.05).

Analysis of variance (ANOVA) was conducted to explore group differences in CU traits. Posthoc comparisons between groups were Sidak-corrected. To investigate the modulation of ERP parameters by CU traits, participants were grouped according to diagnostic status to create dummy variables, as follows: ASD group (ASD/ASD + ADHD versus TD/ADHD); ADHD group (ADHD/ASD + ADHD versus TD/ASD). Separate hierarchical regression analyses were conducted with each ERP parameter (Cue P3, NoGo P3 and the difference in N2 amplitude between Go and NoGo trials, or GoNoGo-N2) entered as the dependent variable. The first block contained group as a predictor (dummy variables for ASD and ADHD as defined above), to recreate previous findings with the same sample (Tye et al., 2014). In the second block, CU traits were added into the model. In the final block, a group*CU traits interaction was added. In the supplementary materials, three additional analyses were conducted, whereby (1) age was added to the second block; (2) scores on the SDQ Conduct sub-scale (see Table 1) were added to the second block to investigate the effect of conduct problems on the relationship between ASD/ADHD, CU traits, and ERP-indexed executive function; and (3) IQ was added to the second block to consider the role of general cognitive ability. The main pattern of results was retained when age, conduct problems and IQ were entered (see Supplementary Material).

## 3 Results

### 3.1 Profile of CU traits

Elevated CU traits were demonstrated in the clinical groups compared to the typically developing children (see Table 1). A significant effect of group on the ICU sum score emerged [F (3, 86) = 31.93, p < 0.001]. Post-hoc tests indicated typically developing children had significantly lower CU traits compared to ASD (p = 0.001), ADHD (p < 0.001) and ASD + ADHD (p < 0.001). Children with ASD + ADHD had significantly higher CU traits compared to ASD (p < 0.001), with a trend towards a difference with ADHD (p = 0.08).

### 3.2 Association between CU traits and ERP parameters

#### 3.2.1 Cue P3: attentional orienting

In block one, we recreated the previous effect using the same sample as Tye et al. (2014a): ADHD diagnosis significantly predicted reduced Cue-P3 amplitude (ADHD/ASD + ADHD vs. ASD/TD; beta = −0.303, p = 0.005), whereas the effect of ASD did not reach significance (ASD/ASD + ASD vs. ADHD/TD; beta = −0.185, p = 0.083). In block 2, the effect of ADHD diagnosis on the Cue P3 became non-significant after accounting for CU traits (beta = −0.190, p = 0.167). No significant associations with ASD (beta = −0.122, p = 0.292) or CU traits (beta = −0.195, p = 0.193) were found. In the final block, the interactions for ADHD by CU traits (beta = 0.711, p = 0.137) and ASD by CU traits (beta = −0.276, p = 0.461) were not significant.

#### 3.2.2 NoGo P3: inhibitory processing

As previously reported (Tye et al., 2014a), ADHD diagnosis (beta = −0.326, p = 0.003) but not ASD diagnosis (beta = −0.046, p = 0.670) significantly predicted reduced NoGo-P3 amplitude. The effect of ADHD diagnosis on NoGo-P3 amplitude remained significant when CU traits were entered into the model (beta = −0.301, p = 0.035). Neither CU traits (beta = −0.043, p = 0.782) nor ASD diagnosis (beta = −0.032, p = 0.787) significantly predicted NoGo-P3 amplitude. There was no significant interaction between ADHD and CU traits (beta = 0.156, p = 0.751), nor for ASD by CU traits (beta = 0.441, p = 0.257).

#### 3.2.3 Go vs NoGo N2: conflict monitoring

**Fig. 1:**
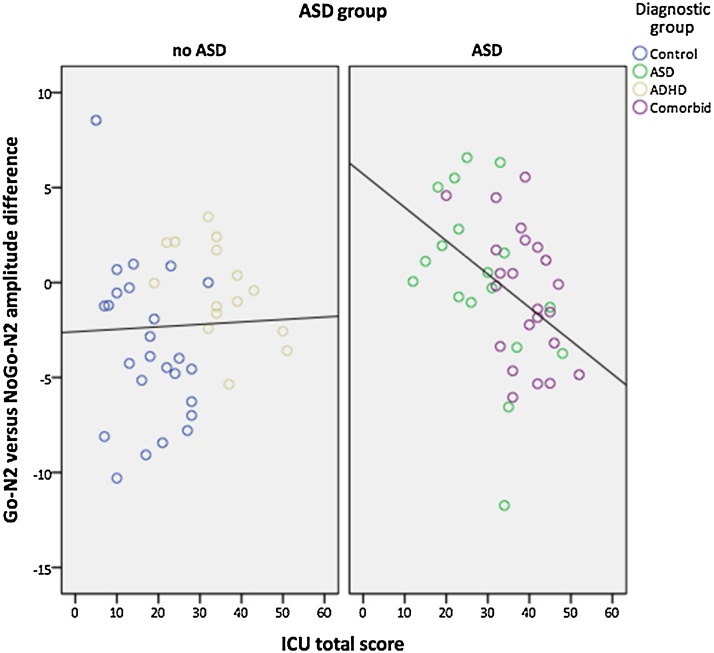
Interaction between ASD diagnosis and CU traits on the Go-to-NoGo-N2 amplitude difference. In the ASD group (children with ASD and comorbid ASD + ADHD), higher CU trait scores are associated with an enhanced Go-NoGo N2 amplitude difference, with greater N2 amplitude towards NoGo compared to Go stimuli. Conversely, children with ASD and low CU trait scores demonstrated greater N2 amplitude towards Go compared to NoGo stimuli. In the no ASD group (children with ADHD and typically developing children), there was no association between CU traits and the Go-NoGo N2 amplitude difference.

As previously shown (Tye et al., 2014a), block 1 showed no significant association between ADHD diagnosis and Go-NoGo-N2 amplitude difference (beta = 0.130, p = 0.245), but ASD diagnosis did significantly predict the amplitude of the Go-NoGo-N2 difference (beta = 0.224, p = 0.046). However, when CU traits were added into the model in block 2, the relationship between ADHD and the Go-NoGo-N2 amplitude difference became significant (beta = 0.400, p = 0.004). CU traits significantly predicted the Go-NoGo-N2 amplitude difference (beta = −0.465, p = 0.002), with higher CU traits associated with greater N2 enhancement from Go to NoGo trials and the association with ASD diagnosis remained significant (beta = 0.372, p = 0.002) after accounting for CU traits. While there was no significant ADHD by CU traits interaction (beta = −0.273, p = 0.557), there was a significant ASD by CU traits interaction (beta = −0.801, p = 0.029), indicating that for children with ASD, higher CU traits were associated with a greater Go-NoGo-N2 amplitude difference, with greater NoGo-N2 compared to Go-N2 amplitude (see Fig. 1).

## 4 Discussion

Individuals with increased callous-unemotional traits represent a putative subgroup of antisocial behaviour that is associated with persistent antisocial behaviour and may be at the core of psychopathy (Viding, 2004). This preliminary study investigated the potentially advantageous role of CU traits on ERP-indexed EF in a small sample of children with clinically diagnosed ASD, ADHD and co-occurring ASD + ADHD during a cued CPT-OX task. Our previous work in the same sample indicated impaired conflict monitoring in children with ASD (ASD-only/ASD + ADHD), as indexed by a reduced enhancement of N2 amplitude from Go to NoGo trials (Tye et al., 2014a). An important and novel finding emerging from this data is the demonstration that CU traits moderate this association. Our analysis indicated that enhanced conflict monitoring was only demonstrated in ASD in the presence of higher levels of CU traits, as demonstrated by greater N2 amplitude to NoGo compared to Go stimuli. Conversely, children with ASD and low CU traits show greater N2 amplitude to Go compared to NoGo stimuli, a reversal from the typical response. This demonstrates heterogeneity within the ASD groups on conflict monitoring that can be partially accounted for by the presence of CU traits, and highlights the importance of a dimensional approach that measures modulation of cognitive profiles across the distribution of co-occurring traits. As CU traits are a proposed antecedent of adult psychopathy, enhanced conflict monitoring associated with high CU traits is in line with previously reported augmented error monitoring and intact attentional switching associated with psychopathic and antisocial traits in non-ASD populations (Ishikawa et al., 2001, Munro et al., 2007, Bresin et al., 2014). In contrast, however, a previous study of individuals with ASD indicated no association between CU traits and cognitive flexibility, a construct which likely relates to conflict monitoring (Rogers et al., 2006, Leno et al., 2015), yet these findings are based on individuals grouped by high or low CU traits, and on cognitive performance that is unable to measure covert processing of stimuli as captured by the N2. Still, in line with previous research, both CU traits and ASD diagnosis predicted the N2 amplitude difference between Go and NoGo trials, but in opposite directions, which suggests that the cognitive deficits associated with CU traits are not central to ASD. Accordingly, reduced conflict monitoring was related to ASD regardless of CU traits, suggesting this impairment is related to core ASD symptoms.

Our findings indicate that CU traits may provide a possible cognitive strength in some individuals with ASD. For example, elevated CU traits may act as a compensatory mechanism in children with ASD through stronger conflict monitoring skills (Johnson, 2012), although this likely interacts with several factors. Such an augmentation associated with CU traits is consistent with models positing a heightened ability of psychopaths to focus on the explicit requirements of a task (Hiatt and Newman, 2006), thus allowing the individual to be more “effective” in their antisocial goals. Similarly, previous work supports the role of CU traits in reactivity to stimuli (Viding et al., 2012), thus the presence of high CU traits in children with ASD may incur increased reactivity and adaptability to changing demands during the CPT-OX (Sinha et al., 2014). Executive function, particularly in the form of performance/conflict monitoring that is implicated here, is closely linked with adaptive behaviour and self-regulation (Ullsperger, 2006). Our previous work has suggested that children with ASD + ADHD have exacerbated impairments in adaptive function compared to children with ADHD-only (Ashwood et al., 2015). This may suggest that general adaptive functioning is improved in children with ASD and CU traits. Moreover, reduced cognitive flexibility has been associated with restricted and repetitive behaviours in ASD (Lopez et al., 2005), so it will be important to consider the role of CU traits in this aspect of ASD. Longitudinal studies are required to assess the causal nature of these relationships and examine whether increased CU traits predict later functioning and core ASD symptoms.

Regardless of the level of CU traits, children with ADHD demonstrated impaired response inhibition as indexed by reduced amplitude of the NoGo-P3. This suggests that impaired inhibition is tied in with core ADHD symptoms and is evident across high and low levels of CU traits. The effect of ADHD diagnosis on attentional orienting did not remain when accounting for CU traits, which may suggest that some of this variance can be attributed to CU traits. When partialling out CU traits, the association between ADHD diagnosis and N2 amplitude difference between Go and NoGo trials became significant, whereby children with ADHD had reduced conflict monitoring once symptoms associated with CU traits were controlled for. The observation that CU traits do not moderate EF in children with ADHD suggests this effect is specific to ASD diagnosis, which may suggest that these behaviours play a qualitatively different role in the disorders. Still, any interpretations given must be taken with caution due to the small sample size, which warrants independent replication attempts prior to any firm conclusions being made.

Taken together, our study shows that different cognitive profiles emerge in children with CU traits and specific neurodevelopmental disorders, which may have implications for identification of further cognitive strengths and weaknesses and subsequent targeting of specific treatment strategies. Our findings imply that there is a form of ASD that co-occurs with high CU traits, perhaps suggesting multiple causal pathways that lead to distinct cognitive abnormalities. This emphasises the importance of considering CU traits as moderators of EF, and raises several questions for future research to investigate the mechanisms underlying this, following critical replication attempts of this pilot study. First, the aetiological pathways linking CU traits in ASD to specific cognitive abnormalities and anti-social behaviour or psychopathy should be explored in longitudinal designs. In addition, the generalizability of this finding to other clinical populations should be investigated. For example, when controlling for conduct problems, CU traits are associated with reduced trait anxiety (Frick et al., 1999, Pardini et al., 2007). In contrast, conduct problems are significantly associated with increased anxiety (Frick et al., 1999). This suggests a suppressing effect of CU traits on anxiety (Frick et al., 2008), which may be reflected at the neural level. The importance of these moderating effects is supported by the present study, which warrants further exploration in other disorders. In addition, given the proposed role of CU traits on persistent antisocial behaviour, the observation that CU traits positively moderate EF in children with ASD supports their consideration in targeting more effective prevention and treatment efforts, by creating more homogenous subgroups based on co-occurring CU traits and associated strengths and weaknesses (Hawes and Dadds, 2005). In support of treatments targeted to putative subgroups, different treatment responses have been demonstrated in children with CD or ADHD with and without CU traits (Haas et al., 2011).

The current preliminary study is limited by a modest sample size. This may limit statistical power to detect a significant interaction between CU traits and ASD/ADHD, which was the primary effect of interest, and reduce the likelihood of replication, thus the findings should be interpreted with caution. Although reduced power may lead to reduced detection of true effects, the likelihood of a false positive is similar to a large sample (Pickles et al., 2015). The findings provide proof-of-concept for the moderating effect of CU traits on EF in childhood ASD (rather than psychopathic traits in the general population or in offender populations) and warrant further investigation and replication in a larger independent sample. Future research directly comparing groups with ASD/ADHD with and without CU traits is needed, using a measure that can capture individuals above cut-off points to allow a group comparison of individuals with and without elevated CU traits. It also remains unclear whether the findings reflect a general pattern or are specific to the outcomes that were selected. For example, a different pattern may have emerged if the focus were on emotional processing deficits that have been specifically linked to CU traits in children with ASD (Leno et al., 2015). Given that previous findings indicate typical performance in psychopaths when a simple task is administered (Budhani and Blair, 2005), the saliency of stimuli and the domain in question should be examined in relation to the effect of CU traits on ASD/ADHD. In addition, there may be different findings depending on the type of CU behaviour investigated (callous, unemotional, uncaring). For example, within psychopathy for which CU traits are a proposed antecedent, primary psychopathic traits (e.g. low anxiety, social dominance, fearless and callousness) are associated with over-focused attention and/or reduced processing of information peripheral to the focus of attention (Sadeh and Verona, 2008). Likewise, interpersonal affective traits have been related to enhanced error monitoring, while there is a limited association with impulsive-antisocial traits (Bresin et al., 2014). There is likely heterogeneity within the CU construct also, which should be explored further within neurodevelopmental disorders using sensitive EEG measures (Almeida et al., 2014, Anderson et al., 2015). Only males were included in the study in order to reduce heterogeneity and due to the higher ratio of males diagnosed with ASD compared to females. A comparison with female participants may be informative given that previous findings indicate lower CU traits and distinct profiles in females compared to males in both community and clinical samples (Essau et al., 2006; Euler et al., 2015). Still, the majority of previous research on CU traits tends to focus on males, which enables direct comparisons with the current work.

## 5 Conclusions

This is the first study to suggest that high CU traits are associated with an altered neurophysiogical profile in children with ASD. Our findings suggest CU traits provide a relative cognitive strength in conflict monitoring in ASD. The measurement of ERPs that index different cognitive markers may help to parse heterogeneity in neurodevelopment disorders and characterise cognitive strengths and weaknesses within certain subgroups in order to target more specific treatment strategies.

## Conflict of Interest

None.
