# Supplementary material for: Callous-unemotional traits moderate executive function in children with ASD and ADHD: A pilot event-related potential study
Source: Dev Cogn Neurosci. 2017 Jun 13;26:84–90. doi: 10.1016/j.dcn.2017.06.002 (PMC5569583; doi:10.1016/j.dcn.2017.06.002)
Supplement: Supplementary file 1 [file mmc1.docx]

**Supplementary Material**

**Association between CU traits and ERP parameters, controlling for age**

Including age as a covariate did not substantively change the results.

*Cue P3: attentional orienting*

For the Cue P3 attentional orienting component, in block 1 there was no significant effect of age (beta = 0.074, p = 0.480). ADHD diagnosis remained a significant predictor of reduced Cue-P3 amplitude (ADHD/ASD+ADHD vs. ASD/TD; beta = -0.294, p = 0.007), with no effect of ASD diagnosis (beta = -0.195, p = 0.072). In block 2, the main effect of age remained non-significant (beta = 0.080, p = 0.443) and the main effects of ASD, ADHD and CU traits were not significant (p values > 0.202). The interactions between ASD and CU traits, and ADHD and CU traits also remained non-significant (p values > 0.118).

*NoGo P3: inhibitory processing*

Again there was no main effect of age (beta = 0.089, p = 0.409). ADHD diagnosis (beta = -0.32, p = 0.005) but not ASD diagnosis (beta = -0.057, p = 0.599) significantly predicted reduced NoGo-P3 amplitude. The effect of ADHD diagnosis on NoGo-P3 amplitude remained significant when CU traits were entered into the model (beta = -0.287, p = 0.046), but CU traits and ASD were not significant (p values > 0.726). Interactions in the final block remained non-significant (p values > 0.294).

*Go vs NoGo N2: conflict monitoring*

Again, age did not predict Go-NoGo-N2 amplitude difference (beta = -0.131, p = 0.238). ASD (beta = 0.211, p = 0.05) and not ADHD (beta = 0.147, p = 0.190) remained significant. In block 2, ASD, ADHD and CU traits all remained significant (p values < 0.004), and in the final block the ASD by CU traits interaction remained significant (beta = -0.874, p = 0.018), and the interaction for ADHD by CU traits remained non-significant (beta = -0.192, p = 0.679).

**Association between CU traits and ERP parameters, controlling for conduct problems**

Including conduct problems (measured on the SDQ) as a covariate did not substantively change the results.

*Cue P3: attentional orienting*

In block one, we recreated the previous effect using the same sample as Tye et al (2014a): ADHD diagnosis significantly predicted reduced Cue-P3 amplitude (ADHD/ASD+ADHD vs. ASD/TD; beta = -0.277, p = 0.021), whereas the effect of ASD did not reach significance (ASD/ASD+ASD vs. ADHD/TD; beta = -0.172, p = 0.146), as previously reported using analysis of variance. In block 2, the effect of ADHD diagnosis on the Cue P3 became non-significant after accounting for CU traits and conduct problems (beta = -0.276, p = 0.100). No significant associations with ASD (beta = -0.115, p = 0.365), CU traits (beta = -0.196, p = 0.265) or conduct problems (beta = 0.199, p=0.212) were found. In the final block, the interactions for ADHD by CU traits (beta = 0.716, p = 0.188) and ASD by CU traits (beta = -0.496, p = 0.218) were not significant.

*NoGo-P3: inhibitory processing*

ADHD diagnosis (beta = -0.249, p = 0.043) but not ASD diagnosis (beta = -0.028, p = 0.815) significantly predicted reduced NoGo P3 amplitude. The effect of ADHD diagnosis on NoGo-P3 amplitude remained significant when CU traits were entered into the model (beta = -0.477, p = 0.004). Neither CU traits (beta = -0.089, p = 0.605) nor ASD diagnosis (beta = 0.065, p = 0.602) significantly predicted NoGo P3 amplitude. Conduct problems significant predicted NoGo P3 amplitude (beta = 0.463, p= 0.004), with higher conduct problems associated with greater NoGo P3 amplitude. There was no significant interaction between ADHD and CU traits (beta = 0.093, p = 0.863), nor for ASD by CU traits (beta = 0.203, p = 0.612).

*Go vs NoGo N2: conflict monitoring*

Block 1 showed no significant association between ADHD diagnosis and Go-NoGo-N2 amplitude difference (beta = 0.170, p = 0.139), but ASD diagnosis did significantly predict the amplitude of the Go-NoGo-N2 difference (beta = 0.383, p = 0.001). However, when CU traits and conduct problems were added into the model in block 2, the relationship between ADHD and the Go-NoGo-N2 amplitude difference became significant (beta = 0.339, p = 0.030). CU traits significantly predicted the Go-NoGo-N2 amplitude difference (beta = -0.441, p = 0.008), with higher CU traits associated with greater N2 enhancement from Go to NoGo trials and the association with ASD diagnosis remained significant (beta = 0.504, p < 0.001) after accounting for CU traits and conduct problems. Conduct problems did not significantly predict the Go/NoGo N2 amplitude difference (beta = 0.174, p = 0.239). While there was no significant ADHD by CU traits interaction (beta = -0.741, p = 0.110), there was a significant ASD by CU traits interaction (beta = -1.049, p = 0.003), indicating that for children with ASD, higher CU traits were associated with a greater Go-NoGo-N2 amplitude difference.

**Association between CU traits and ERP parameters, controlling for IQ**

Including IQ in as a covariate did not substantively change the results.

*Cue P3: attentional orienting*

For the Cue P3 attentional orienting component, in block 1 there was no significant effect of full scale IQ (beta = 0.054, p = 0.629). ADHD diagnosis remained a significant predictor of reduced Cue-P3 amplitude (ADHD/ASD+ADHD vs. ASD/TD; beta = -0.283, p = 0.015), with no effect of ASD diagnosis (beta = -0.187, p = 0.082). In block 2, the main effect of full scale IQ remained non-significant (beta = 0.047, p = 0.673) and the main effects of ASD, ADHD and CU traits were not significant (p values > 0.203). The interactions between ASD and CU traits, and ADHD and CU traits also remained non-significant (p values > 0.144).

*NoGo P3: inhibitory processing*

Again there was no main effect of IQ (beta = 0.091, p = 0.424). ADHD diagnosis (beta = -0.291, p = 0.014) but not ASD diagnosis (beta = -0.049, p = 0.651) significantly predicted reduced NoGo-P3 amplitude. The effect of ADHD diagnosis on NoGo-P3 amplitude became marginally significant when CU traits were entered into the model (beta = -0.271, p = 0.067), but CU traits and ASD were not significant (p values > 0.756). Interactions in the final block remained non-significant (p values > 0.274).

*Go vs NoGo N2: conflict monitoring*

Again, IQ did not predict Go-NoGo-N2 amplitude difference (beta = -0.120, p = 0.310). ASD (beta = 0.228, p = 0.043) and not ADHD (beta = 0.085, p = 0.480) remained significant. In block 2, ASD, ADHD and CU traits all remained significant (p values < 0.014), and in the final block the ASD by CU traits interaction remained significant (beta = -0.779, p = 0.034), and the interaction for ADHD by CU traits remained non-significant (beta = -0.245, p = 0.598). ***Table S1:***  Mean amplitude (in μV) for ERPs elicited during the CPT-OX by group

|  |  | Diagnosis | | | | | | | | | |
| --- | --- | --- | --- | --- | --- | --- | --- | --- | --- | --- | --- |
|  |  | TDC  (*n* = 25) | | ASD  (*n* = 19) | | | ADHD  (*n* = 16) | | ASD+ADHD  (*n* = 25) | | |
|  |  | Mean | SD | | Mean | SD | Mean | SD | | Mean | SD |
| Cue | P3 (area μV at Pz) | 9.46 | 3.56 | | 7.39 | 4.54 | 6.72 | 3.20 | | 5.77 | 2.76 |
| Go | N2 (peak μV at Fz) | -6.88 | 4.45 | | -7.41 | 5.70 | -5.68 | 4.19 | | -3.60 | 3.85 |
| NoGo | P3 (area μV at Cz) | 6.82 | 4.44 | | 6.81 | 5.64 | 4.13 | 2.33 | | 4.95 | 3.68 |
|  | P3 (area μV at CPz) | 7.47 | 4.63 | | 7.12 | 4.95 | 5.17 | 2.95 | | 3.80 | 2.16 |
|  | P3 (area μV at Pz) | 6.96 | 5.32 | | 5.91 | 3.00 | 4.85 | 3.94 | | 4.22 | 2.96 |
|  | N2 (peak μV at Fz) | -10.28 | 4.73 | | -7.32 | 4.78 | -6.44 | 4.52 | | -4.69 | 3.44 |

**Supplementary Figure legends**

**Figure S1: Grand mean ERPs to Cue stimuli for each group and isocontour maps derived for the grand-average in the 400-700ms window for Cue-P3 for each group, plus t-maps for the group comparison**. Black represents TD, red represents ASD-only; blue represents ADHD-only; green represents comorbid ASD+ADHD.

**Figure S2: Grand mean ERPs to NoGo stimuli for each group and isocontour maps derived for the grand-average in the 400-700ms window for NoGo-P3 for each group, plus t-maps for the group comparison**. Black represents TD, red represents ASD-only; blue represents ADHD-only; green represents ASD+ADHD.
