# Supplementary figures and images for: Callous-unemotional traits moderate executive function in children with ASD and ADHD: A pilot event-related potential study
Source: Dev Cogn Neurosci. 2017 Jun 13;26:84–90. doi: 10.1016/j.dcn.2017.06.002 (PMC5569583; doi:10.1016/j.dcn.2017.06.002)

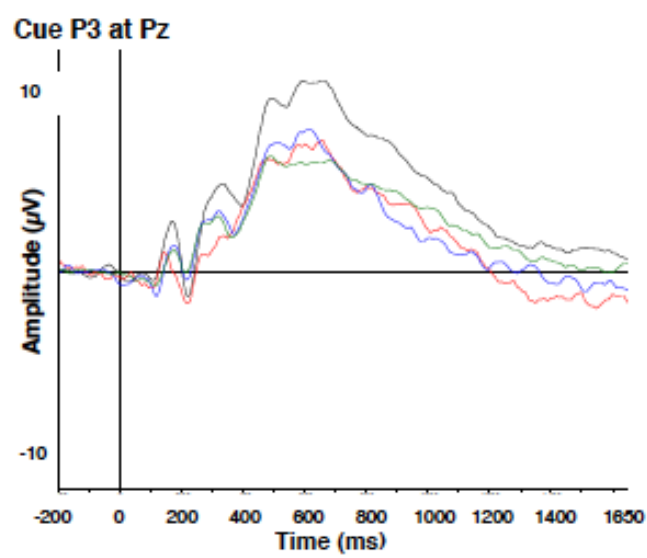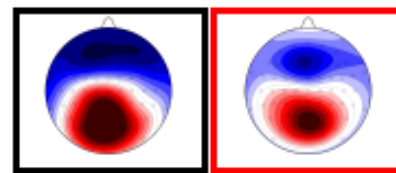

TD

ASD

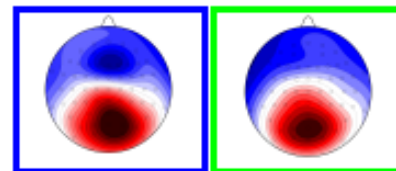

ADHD

ASD+ADHD

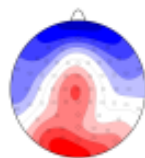

TD vs ASD

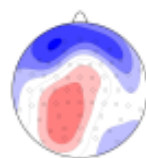

TD vs ADHD

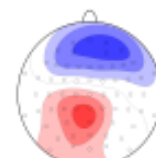

TD vs ASD+ADHD

Supplement: Supplementary file 2 [file mmc2.pdf]

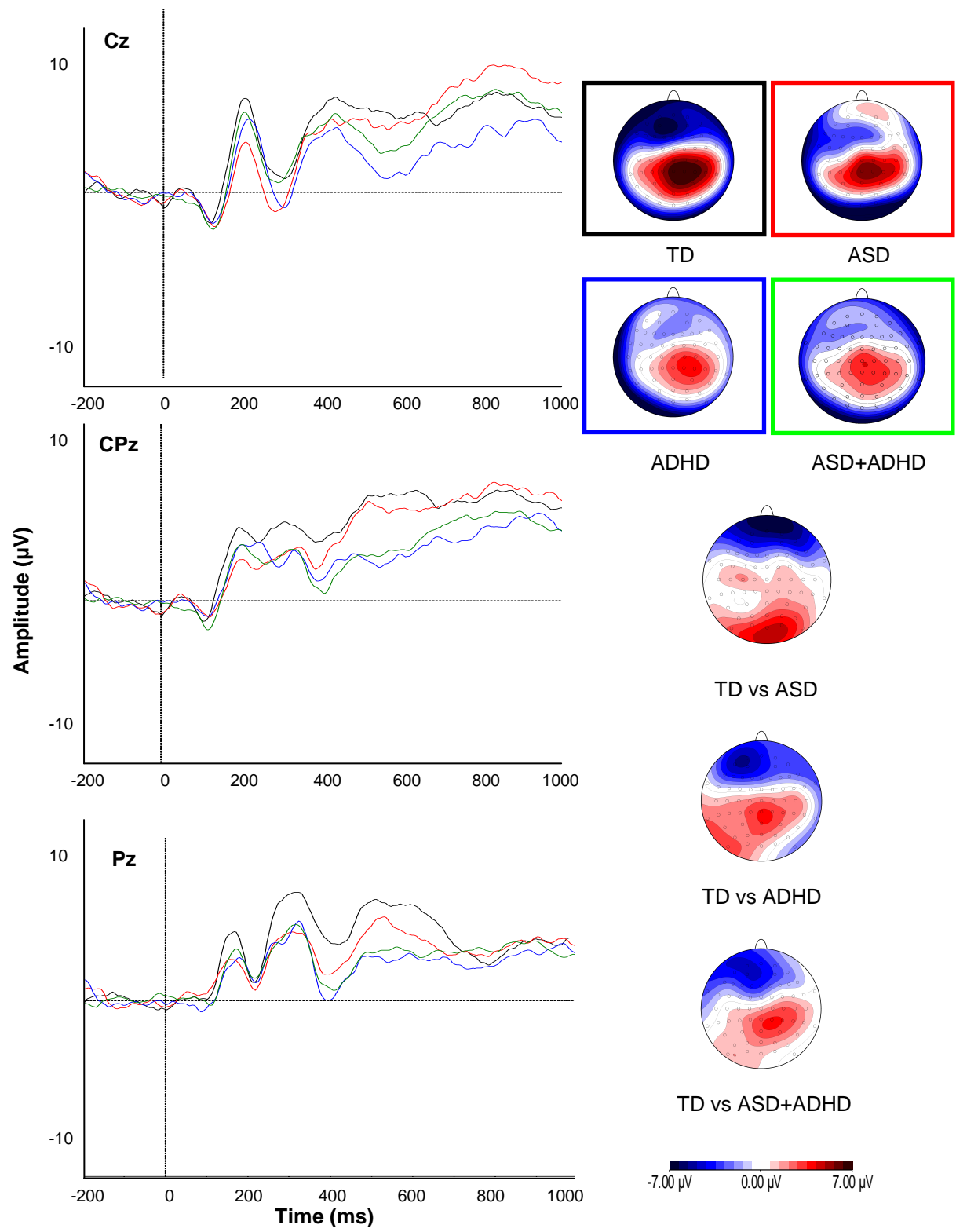

Supplement: Supplementary file 3 [file mmc3.pdf]
